# Supplementary material for: Extracting the multi-timescale activity patterns of online financial markets
Source: Sci Rep. 2018 Jul 25;8:11184. doi: 10.1038/s41598-018-29537-w (PMC6060124; doi:10.1038/s41598-018-29537-w)
Supplement: Supplementary file 1 — Supplementary Information [file 41598_2018_29537_MOESM1_ESM.pdf]

Supplementary Information

"Extracting the multi-timescale activity patterns of online financial markets"

Teruyoshi Kobayashi, Anna Sapienza and Emilio Ferrara

S1 Temporal heterogeneity of bank activity

In Fig. S1, we illustrate the heterogeneity of bank activity at inter- and intraday scales. We pick eight banks whose number of participated days are ranked 1st, 10th, 30th, 50th, 80th, 100th, 150th and 200th. The banks' activities are measured by their total volumes of transactions within a given day or a given time interval.

S2 Intra- and inter-day activity of banks

The r-th rows of factor matrices $\mathbf{B}$ and $\mathbf{C}$ (i.e., $\mathbf{b}_r$ and $\mathbf{c}_r$) respectively represent the intra- and inter-day activities of component r. Fig. S2 illustrates intra- and inter-day activities for a given temporal resolution $\Delta \in \{5, 30, 45\}$. The selected rank size, based on the Core-Consistency measure, is $R = 4$ for $\Delta = 5$, $R = 3$ for $\Delta = 30$, and $R = 2$ for $\Delta = 45$. We note that the choice of Δ does not affect the activity distributions for a fixed value of R.

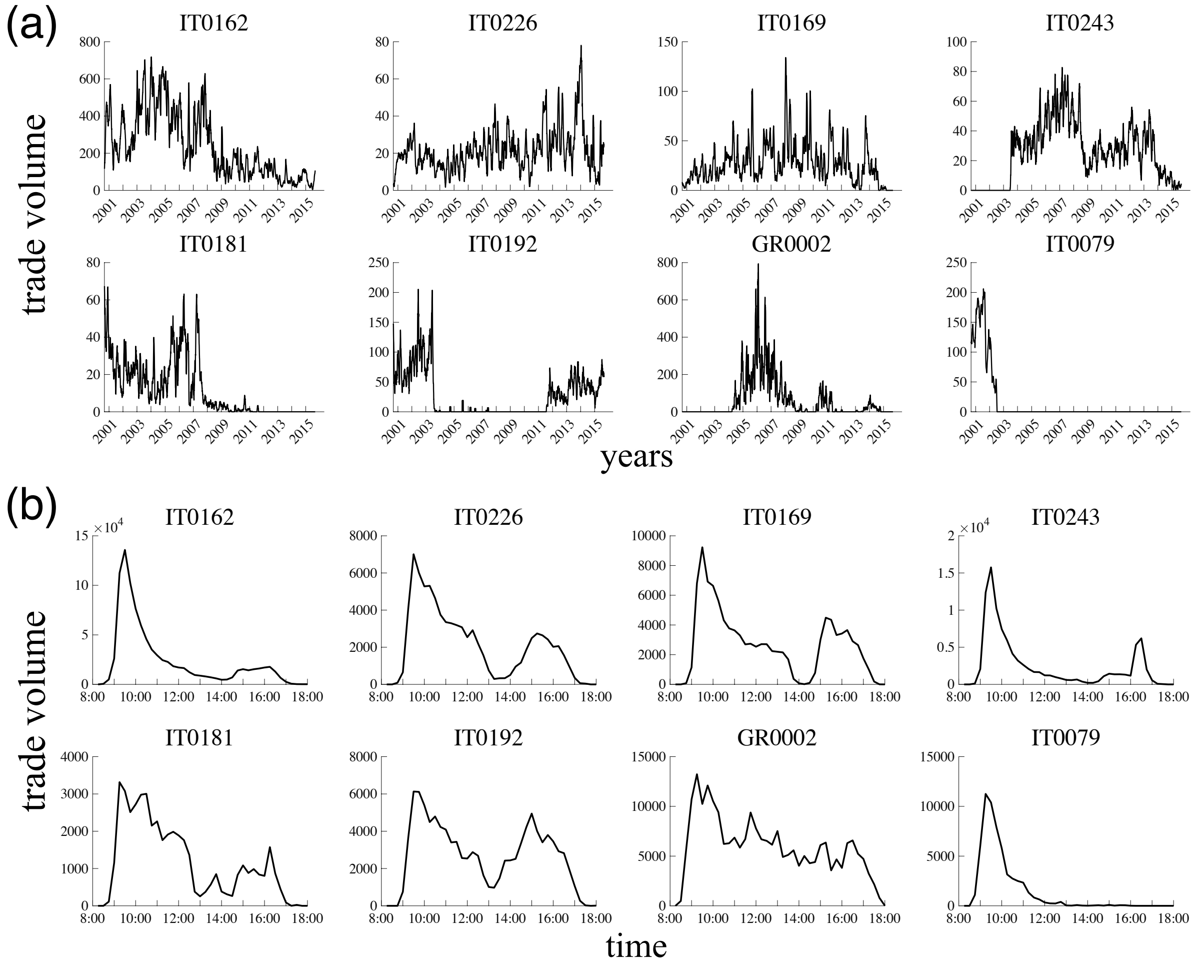

Figure S1: Bank activity at interday and intraday scales. Bank ID is annotated at the top of each panel. (a) 20-day moving average for the total volumes of transactions (in million Euros) conducted in each day. (b) Total amount of transactions conducted by a bank in each time interval (i.e., 15 min).

(a) $\Delta = 5$

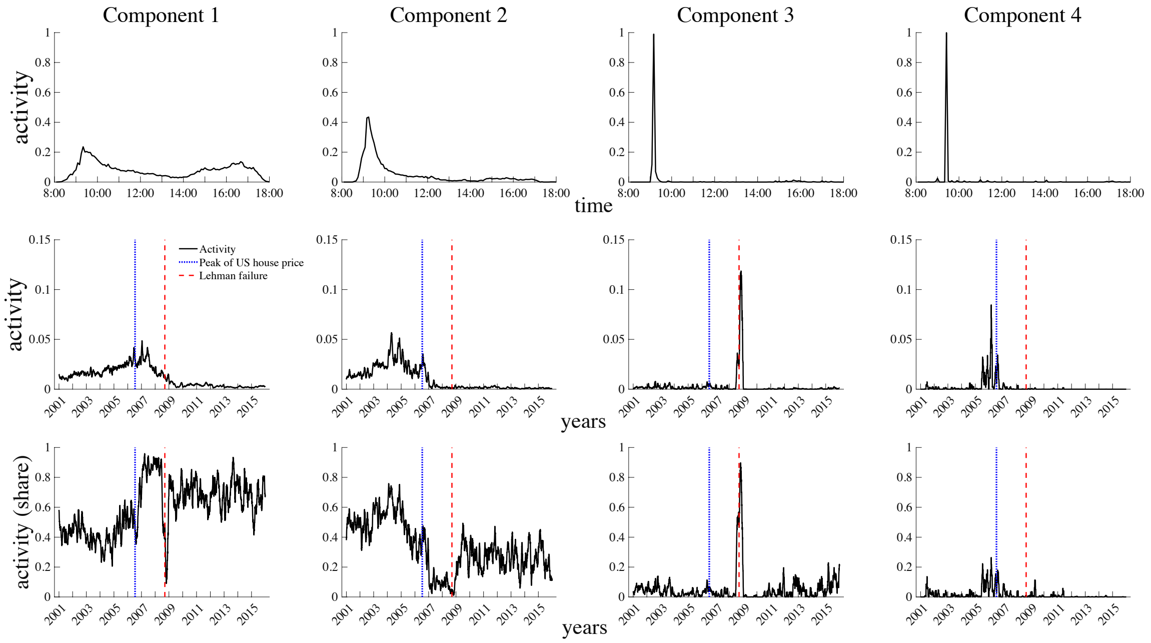

Figure S2: Intra- and inter-day activities. Upper: Intra-day activity. Middle: Inter-day activity. Lower: Share of a component in the daily activity. (a) $\Delta = 5$, (b) $\Delta = 30$, and (c) $\Delta = 45$. See the caption of Fig. 7.

(b) $\Delta = 30$

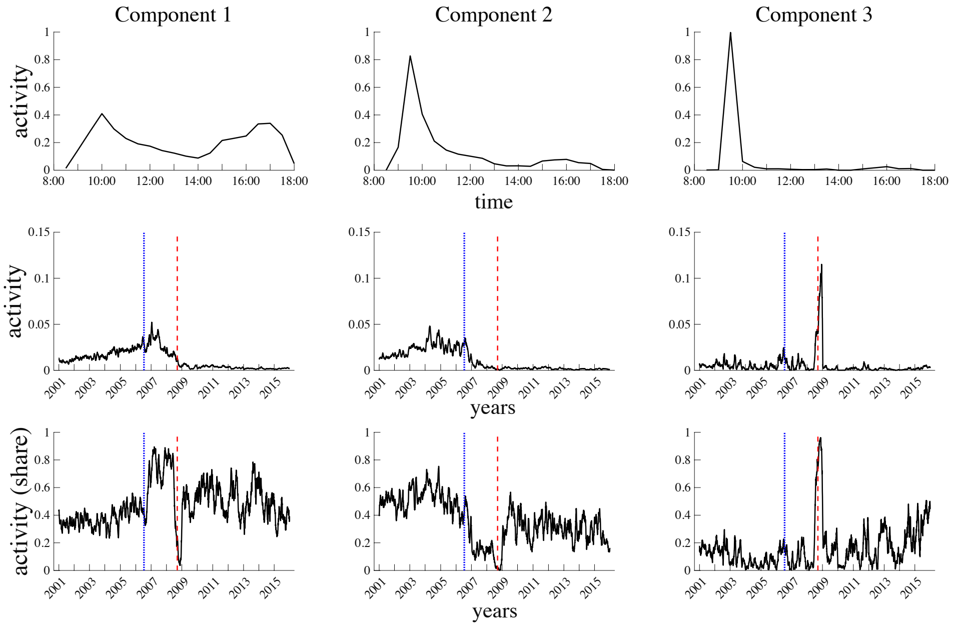

(c) $\Delta = 45$

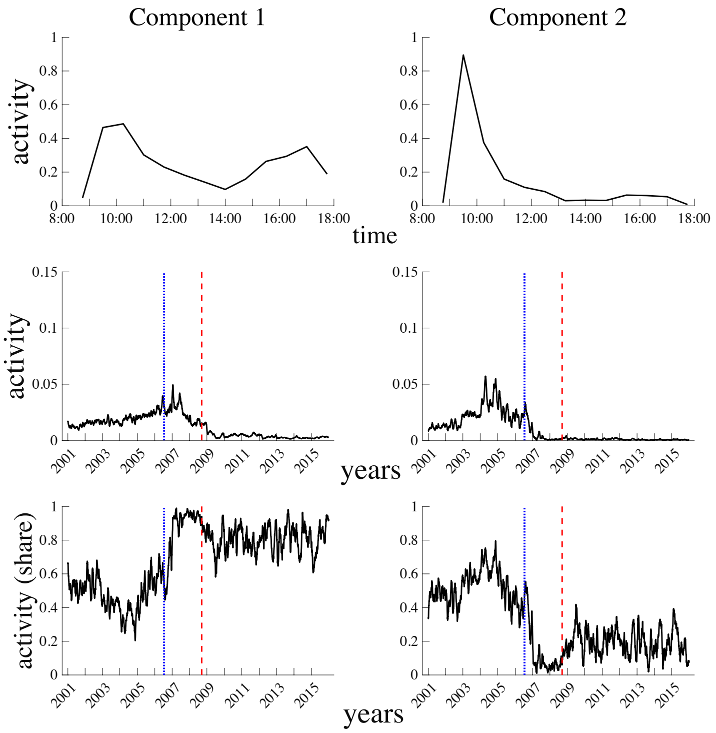
Component 1
Component 2
activity
time
8:00
10:00
12:00
14:00
16:00
18:00
activity
years
2001
2003
2005
2007
2009
2011
2013
2015
activity (share)
years
